# Physicochemical properties of a new structural lipid from the enzymatical incorporation of flaxseed oil into mutton tallow

**DOI:** 10.1016/j.heliyon.2022.e09615

**Published:** 2022-06-04

**Authors:** Jun Liu, Weiyi Zhang, Dunhua Liu, Wei Zhang, Lu Ma, Shuzhe Wang

**Affiliations:** a School of Agriculture, Ningxia University, 750021, Yinchuan, Ningxia, China; b School of Modern Agriculture & Biotechnology, Ankang University, 725000, Ankang, Shanxi, China; c School of Food & Wine, Agricultural Product Analysis and Safety Laboratory, Ningxia University, 750021, Yinchuan, Ningxia, China; d Guyuan Branch of Ningxia Academy of Agricultural and Forestry Sciences, 756000, Guyuan, Ningxia, China; e Department of Business Management, Shizuishan Institute of Industry and Trade, 753000, Shizuishan, Ningxia, China

**Keywords:** Structured lipid, Transesterification, Fatty acids, Rheological properties, Volatile compounds

## Abstract

This study evaluated the physio-chemical properties of a structural lipid (SL) obtained by the enzymatical incorporation of flaxseed oil into mutton tallow (MT). By measuring the melting point, colour, safety, fatty acids, apparent viscosity, shear stress and volatile compounds of the samples, the results showed that compared to MT, SL exhibited lower L∗(lightness) value, melting point, apparent viscosity and shear stress (*p* < 0.05). Noteworthy, the Saturated fatty acids (SFA)content of MT was reduced from 61.46% to 25.49% (*p* < 0.05), although SL was found to be more prone to oxidation during storage. The clearest discrepancy in volatile compounds was the increase of heterocyclic compounds in SL. In summary, improving the edible properties of animal fats by adding vegetable oils is an effective solution, and SL may have a great potential to be developed into a high-quality product with improved nutritional composition of animal fat.

## 1 Introduction

Interesterification is the exchange of fatty acids (FA) within and between the triacylglycerol (TAG) fractions, resulting in the production of structured lipids (SL). These specialized lipids can be designed to contain desired fatty acid compositions with a variety of applications, to variety of application in pharmaceutical and food industry [1]. Although the modification of animal fat has been studied, it is a considerable technical challenge to improve the edible property of animal fats by adding vegetable oils [2]. By using the specific properties of lipases, enzymatic biosynthesis of FA lipids has been used as an attractive alternative to the conventional chemical processes. Enzymatic transesterification has been utilized to improve cheap and saturated fats or increase the value of commercial fats. For example, beef tallow was transesterified with rapeseed oil [3] and with canola oil to produce fat compounds with improved melting properties [4]. These are low-calorie and dietary structured lipids suitable for obesity control and also for people with impaired fat absorption and other metabolic problems. These modified SL are low-calorie and have more rational FAs. Therefore, they are more suitable for special groups of people, for example, those who are obese, have impaired lipid digestion and other disorders of the metabolic system. The esterification process, which has different lengths of fat chains, is more likely to produce medium-chain fatty acids (MCFA), which, due to the specific hydrolysis of the sn-1,3 positions in these MCFA by pancreatic lipase, result in rapid absorption and transport of these MCFA, which then participate in energy metabolism in the liver and do not accumulate as fat in the body [5].

Mutton tallow (MT) is composed of 62% of saturated fatty acids of which more than 57% are MCFA, such as C16:0 and C18:0. Intake of dietary saturated fatty acids has been well-known to be associated with increasing low-density lipoprotein cholesterol, thus, associated with increasing the risk of cardiovascular diseases [6]. MT is conventionally disposed of as waste materials and used for non-edible or industrial products in China. Several studies have reported the potential use of mixing vegetable oils with animal-derived fats in various food products [6, 7]. Flaxseed oil (FO), known to be the richest source of the n-3 fatty acids, can be used to selectively incorporate the desired acyl moiety onto a specific position of the TAG of MT through enzyme-catalyzed reactions. The use of lipases with specific esterification for the sn-1,3 position in the FA chain binds FO to MT. These properties can be modified by preparing SL, which can be used more widely in foods [4].

This study aimed to develop and characterize MT substitutes by constructing healthier animal fat blends. The results of the study may help determine the feasibility of using enzymatic interesterification to produce structured lipid (SL) with balanced fatty acid composition and modified physical and chemical properties. Our results have shown that SL may have a great potential to be developed into a high-quality product with improved nutritional composition of animal fat.

## 2 Materials and methods

### 2.1 Materials

FO and MT were purchased from Ningyang supermarket in Yinchuan, Ningxia, China, and Novozym 435 (Aspergillus oryzae fermentation, Enzyme activity 10,000 PLU/g, optimum temperature 55 °C) was from Novozymes Biotechnology Co., Ltd (Bejing, China). Hexane was of liquid chromatography grade. Other chemicals and reagents not specifically described in this study are of analysis pure reagent.

### 2.2 Preparation of SL

SL samples were prepared as described by the previous authors with a slight modification [8]. Lipase-catalyzed transesterification reaction of MT and FO was performed in a 50 mL conical flask. A representative reaction mixture contained 30 g of MT and 10 g of FO (3:1, m/m). The transesterification reaction was started by the addition of 25 mg of the solid lipase into the conical flask and stirring in a continuous water bath for 8 h (temperature 55 °C, 150 rpm). After the reaction, the lipase was removed by centrifugation at 10,000x g for 15 min, and the crude product of SL was obtained by filtration passing through Whatman 42 filter paper. To obtain the required concentration, 60 mL n-hexane was added to the crude product, mixed with 20 mL 0.5 M KOH solution for alkali refining and deacidification. The dry product was obtained by reduced pressure concentration (vacuum level 0.08–0.09 Mpa, water bath temperature 65 °C) through a rotary evaporator (RE-5220A, Shanghai Yarong Biochemical Instrument Factory, Shanghai, China) and finally by vacuum freeze-drying (cold hydrazine at -40 °C, vacuum at 60 Pa, drying compartment at 25 °C).

### 2.3 Color analysis

The color of the sample was measured with Commission International Eclairage (CIE) for L∗(lightness), a∗ (redness) and b∗ (yellowness) using a CR400 Minolta colorimeter (Konica Minolta Inc., Tokyo, Japan). The light source of C with an aperture of 8 mm and the additional closed cone was calibrated utilizing a white plate (x = 0.3135, y = 0.3198, Y = 88.3). The whiteness was calculated utilizing the following formula:

$$100-\sqrt[{2}]{{\left(100-{\text{L}}^{\text{∗}}\right)}^{2}+\left({\text{a}}^{2}\times {\text{b}}^{2}\right)}$$

The sample was poured into a glass bottle, and the color of the sample was evaluated at 5 separate positions against a standard whiteboard background [9, 10].

### 2.4 Melting point measurement

A 2 mm diameter capillary tube was immersed into the melted oil sample to be tested until the oil in the tube reached a height of 10 mm. The tube was removed, placed quickly into liquid nitrogen to cool and solidify, and freezed in a refrigerator (−18 °C to -20 °C) for 12 h. The capillary tubes were then put into a water bath (ramp-up procedure: 0.1 °C/min), and the temperature of the fat column sliding upwards in each capillary tube was recorded.

### 2.5 Methylation and gas chromatography and mass spectrometry (GC-MS) analysis

GC-MS was performed as reported by the previous authors with minor modifications [8, 11]. The reaction mixture (400 mg) was compounded with hexane (6 mL), phenolphthalein solution (6 mL) and 0.5 M KOH solution (3.2 mL) in ethanol (20%). After shaking, the upper liquid layer was collected, the lower liquid layer was again washed with hexane (2 mL), the upper liquid layer was again collected, and then combined with SL (1.2 mL) and saturated NaCl solution (2.4 mL). After being shaken again, the hexane was collected and evaporated to acquire the deacidified reaction product (DRP); the samples were then sealed using parafilm and stored at 4 °C for analysis within one week.

One microliter of fatty acids (FA) methyl esters was injected into an ODS-SP chromatographic column (4.6 × 250 mm, 5 μm) (Shimadzu Inc., Tokyo, Japan) with the separation mode of 1:12. The temperature program of the column was as follows: 2 min at 180 °C, ramp up to 230 °C at a rate of 3 °C/min and finally held at 230 °C for 10 min, and the temperatures of the injector and detector were 250 °C. The detection voltage was 350 V, and the carrier gas was He_2_ (≥99.99%). MS conditions were as follows: EI ion source, emission current 200 amu, scan range 20–550 amu and electron energy 70 eV. Identification of FAs by comparison of retention times and FAME criteria and the relative contents expressed as the weight percentage (%, *w/w*) were calculated. Each sample was analyzed three times.

### 2.6 Oxidative stability

The acid value, peroxide value and saponification value of the oils and fats were determined by cold solvent indicator titration, KOH–C_2_H_5_OH boiling method [12, 13]. The samples were stored in 500 ml PET bottles (polyethylene terephthalate) at 60 °C and 50 ± 10% relative humidity for 14 days [14]. The samples (1 g) were placed in a 10 mL flask, mixed with acetic acid and chloroform solution (3:2, *v/v*), and 1 mL of saturated potassium iodide solution was added. After the flask was away from light for 10 min, deionized water (30 mL) was added. The sample solution was titrated against 0.01 M sodium thiosulphate solution, with 1% starch solution (1 mL) as the indicator. The peroxide value was expressed as milliequivalents of active oxygen per kg of samples (meqO_2_/kg).

### 2.7 Rheological properties

The rheological properties of FO, MT and SL were measured using an AR1500ex rheometer (TA Instruments (Shanghai) Co. New Castle, USA). The procedure was set up with reference to the method of the previous authors [15], the linear sweeps were performed using a parallel plate geometry system (diameter = 40 mm), with set gap of 1000 μm, fixed angular frequency of 10 rad/s, at 55 °C with shear rates ranging from 0 s^−1^ to 400 s^−1^. To test the effect of temperature (55–95 °C) on viscosity and shear stress, the shear rate and heating rate were set at 50 s^−1^ and 5 °C/min respectively.

### 2.8 Volatile compounds (VCs) analysis

As reported by the previous authors with minor modifications [16]. To extract volatile compounds, 10 g of MT, FO and SL were put into a headspace bottle. The sample vial was then capped securely with a PTFE-silicon stopper. Afterward, the sample vial was equilibrated at 50 °C for 20 min on a heating agitation platform. The extraction was performed by inserting the pretreated SPME fibers (50/30 μm divinylbenzene/carboxen on poly (dimethylsiloxane) DVB/CAR/PDMS), into the headspace of the vial for 40 min, with continuous heating and agitation. When extraction was finished, the fiber was desorbed into the GC injection port for 5 min [16].

The organic phase was analyzed using gas chromatography (GC-2010, Shimadzu Inc., Kyoto, Japan), with the DB-WAX chromatographic column (30 m × 0.25 μm, 0.5 μ MDF) (Shimadzu Inc., Tokyo, Japan). The extraction head was inserted into the sample inlet, and the fiber head was pulled out and analyzed for 5 min. Oven temperature was kept at 60 °C for 2 min initially, then increased to 100 °C at a rate of 3 °C/min and held for 5 min, then from 100 to 180 °C at 5 °C/min and held for 5 min, and reached a final temperature of 230 °C at a rate of 8 °C/min and held for 10 min. Mass specialty conditions were ionization mode EI, ion source temperature 200 °C, electronic energy 70 eV, filtration emission current 200 μA, interface temperature 250 °C and scanning mass range (32–402 amu).

### 2.9 Statistical analysis

The results were expressed as mean ± standard deviations (SD). The figures and principal component analysis (PCA) were plotted with Origin 2018b for Windows (OriginLab Inc., Northampton, USA). Data were analyzed by one-way ANOVA, followed by Duncan's multiple range test using SAS 8.2 for Windows (NCSU, NC State, Raleigh, North Carolina, USA). *P* < 0.05 was considered to be statistically significant.

## 3 Results and discussion

### 3.1 Physicochemical properties

The differences in the melting points reflect the existing state and edible quality of oils and the differences in the fatty acid compositions. FO showed the characteristics of typical vegetable oil, with a lower melting point of -25.3 ± 0.9 °C (Table 1). MT showed the characteristics of typical animal fat, with a higher melting point (46.1 ± 1.1 °C). Compared with MT, the melting point of SL (−6.0 ± 1.0 °C) was lower (*p* < 0.0001), indicating that the prepared SL can effectively improve the edible quality of MT. The change in the melting point may be mainly caused by the changes in the contents of triglyceride with a high melting point, medium melting point and low melting point in the mixture [17].

**Table 1:** The physicochemical properties of FO, MT and SL.

Physicochemical properties	FO	MT	SL
Melting point (°C)	-25.3 ± 0.9^a^	46.1 ± 1.1^b^	-6.0 ± 1.0^c^
L∗	18.93 ± 0.04^a^	40.50 ± 0.40^b^	18.84 ± 0.02^a^
a∗	1.80 ± 0.03^a^	-2.39 ± 0.01^b^	2.12 ± 0.02^c^
b∗	-0.11 ± 0.01^a^	6.20 ± 0.10^b^	6.20 ± 0.10^b^
Acid value (mg/g)	1.26 ± 0.029^a^	0.780 ± 0.006^b^	0.85 ± 0.026^c^
Peroxide value (g/100g)	0.80 ± 0.001^a^	2.730 ± 0.002^b^	1.130 ± 0.001^c^
Saponification value (mg/g)	129.2 ± 0.8^a^	227.3 ± 0.9^b^	184.0 ± 1.0^c^

Note: a. b. c. (→) Different letters within a row indicate a significant difference (*p* < 0 .05). All values are the mean ± standard deviations of three replicates.

There were significant differences between SL and MT in L∗, a∗ and b∗ values (*p* < 0.05), indicating that SL and MT have great differences in color (Table 1). The value of L∗ in SL was lower than MT, but the value of b∗ in SL was higher than MT, and visual perception showed the brightness of SL. The three oils and fats had significantly different colors due to fat-soluble pigments. For example, carotenoids and flavonoid pigments gave FO a reddish-brown color. SL was light yellow, as the process of transesterification may cause carotenoids, flavonoid pigments and other pigments to be oxidized and decomposed [18].

There were significant differences in oxidative stability values of FO, MT and SL among all samples (Table 1). The physicochemical indexes of all these oils and fats were lower than the maximum value reported by Codex Alimentarius (2005). The acid value, peroxide value and saponification value of MT changed from 0.780 ± 0.006 mg/g, 2.730 ± 0.001 g/100g and 227.3 ± 0.9 mg/g to 0.85 ± 0.02 mg/g (*p* < 0.0098), 1.130 ± 0.001 g/100g (*p* < 0.0214) and 184 ± 1 mg/g (*p* < 0.0001).

### 3.2 Fatty acids

As shown in Table 2, the type and proportion of FAs were significantly different among the samples. Saturated fatty acids (SFA, 61.50 ± 0.91%) were predominant in MT, followed by monounsaturated fatty acids (MUFA, 33.14 ± 0.28%) and polyunsaturated fatty acid (PUFA, 1.00 ± 0.09%) (*p* < 0.002). In contrast, PUFA (50.09 ± 1.06%) was predominant in SL, followed by SFA (25.51 ± 0.22%) and MUFA (23.17 ± 0.08%) (*p* < 0.0004). Stearic acid (C18:0) was the predominant SFA in all the samples, followed by palmitic acid (C16:0). Oleic acid (C18:1) was the predominant MUFA in all the groups. Concerning PUFA, of note was the new production of eicosatrienoic acid (C20:3 8,11,14) (41.63 ± 0.92%) in SL.

**Table 2:** The FAs composition of triacylglycerols in FO, MT and SL (expressed as a percentage of total FAs).

FAs	FO (%)	MT (%)	SL (%)
SFAs	13.06 ± 0.04^aA^	61.50 ± 0.91^aB^	25.51 ± 0.22^aC^
Myristic acid (C14:0)	ND	2.28 ± 0.04	0.99 ± 0.03
Pentadecanoic acid (C15:0)	ND	0.49 ± 0.02	ND
Palmitic acid (C16:0)	5.51 ± 0.12	20.13 ± 0.15	10.31 ± 0.09
Margaric acid (C17:0)	7.52 ± 0.07	1.5 ± 0.1	0.87 ± 0.09
Stearic acid (C18:0)	ND	37.1 ± 0.7	13.7 ± 0.2
MUFA	0^bA^	33.14 ± 0.28^bB^	23.17 ± 0.08^bC^
Oleic acid (C18:1)	ND	33.14 ± 0.28	23.17 ± 0.08
PUFAs	87.44 ± 0.15^cA^	1.00 ± 0.09^cB^	50.09 ± 1.06^cC^
Linoleic acid (C18:2)	18.89 ± 0.04	1.00 ± 0.09	6.90 ± 0.12
Linolenic acid (C18:3)	68.5 ± 0.1	ND	1.65 ± 0.05
Eicosatrienoic acid (C20:3 8,11,14)	ND	ND	41.63 ± 0.92

Note: (1) a. b. c (↓) Different letters within a column indicate a significant difference (*P* < 0 .05). (2) A. B. C. (→) Different letters within a row indicate a significant difference (*P* < 0 .05). (3) All values are the mean ±standard deviations of three replicates. (4) ND, non-detected.

SL was produced by the transesterification of FO and MT using lipase. The enzymatic catalytic site and activity of lipases were found to result in differences in the extent of acyl migration [4]. As expected, the FA composition of MT was changed significantly after interesterification. The result showed the variety in the composition and positional distribution of FAs in the triacylglycerols, a decrease of the SFAs (C16:0 and C18:0), MUFA (C18:1) in MT, and the formation of new FA (C18:3, C20:3 8,11,14), due to redistribution of FAs by lipase. After modification, C20:3 8,11,14 became the major FA in SL (Table 2). The presence of USFA bonds at the sn-2 position in SL meant that acyl shift arose during lipase-catalyzed hydrolysis.

### 3.3 Oxidative stability

**Figure 1:**
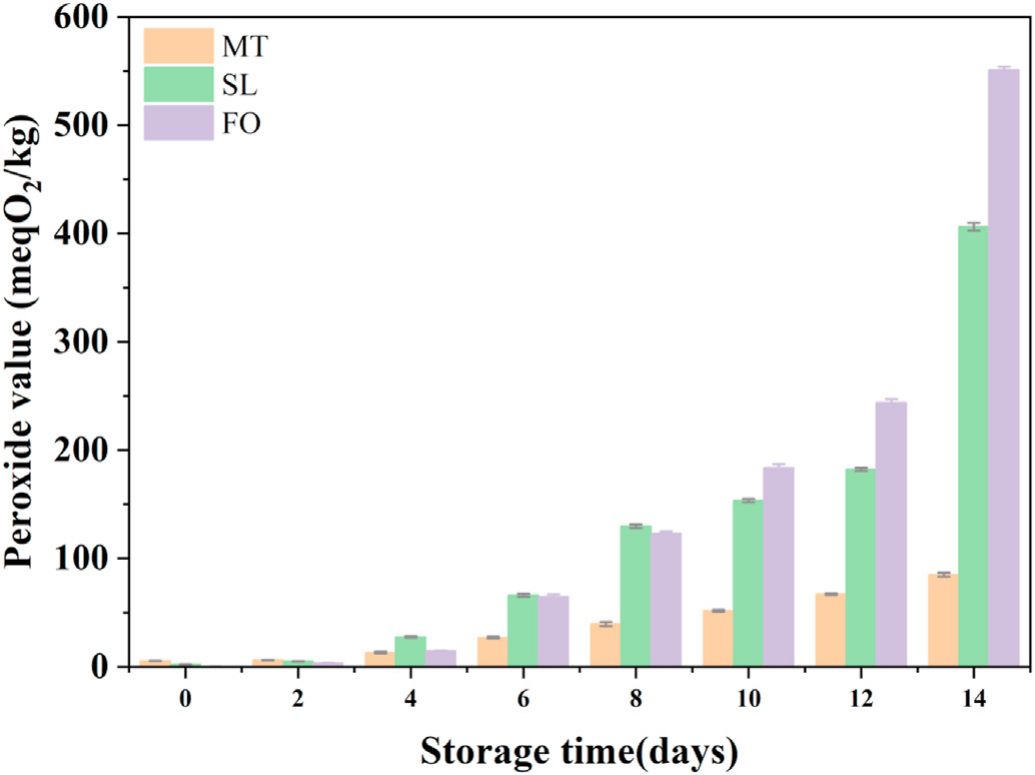
Oxidative stability of FO, MT and SL under storage conditions at 60 °C for 14 d.

The peroxide values of the three samples were monitored for 14 days at 60 °C to investigate the effect of lipase-catalyzed transesterification on the oxidative stability of the SL. The peroxide values of the three samples tended to increase with an increase in the storage time (Figure 1). After the 14-day storage at 60 °C, FO had the highest peroxide value (551 ± 3 meqO_2_/kg), followed by SL (406 ± 4 meqO_2_/kg) and MT (85 ± 2 meqO_2_/kg). The peroxide values of FO, MT and SL seemed to be too high, which may be because they were stored at a high temperature (65 °C) (Codex Alimentarius, 2005). Notably, the increased rate of the peroxide value in SL was significantly lower than that in FO, and the increased rate of the peroxide value in MT was significantly lower than those in FO and SL. The growth rate of FO peroxide value was lower than that in SL in 0–8 days but higher than that in SL in 8–14 days. There is evidence that the loss of nutrients, shortened shelf life, off-flavours and discolouration of lipids are closely linked to lipid oxidation. However, as the primary stage of lipid oxidation ends, deeper oxidation of lipids leads to the accumulation of lipid peroxides (e.g. ketones and aldehydes), which are potentially toxic and carcinogenic [19]. The hydroperoxide from fatty acid generated in the early stage of oil and fat oxidation is the key product leading to the oxidation and rancidity of oil and fat, as it is very unstable [20]. It can further decompose into aldehydes, ketones, and other oxides during the oxidation process, which further deteriorates the oil and fat quality. Therefore, peroxide value can be used to measure the primary oxidation degree of oil and fat. The higher free FA contents of FO and SL have been reported to increase the peroxide value of oils and fats owing to the pro-oxidant effect of the carboxylic groups in FAs [21, 22]. This may be because the content of saturated FA in MT was the highest, but it still contained 34.05% USFA, which may be oxidized under the action of light and heat. However, FO contains a large number of USFA and some reducing active substances, such as fat-soluble polyphenols, flavonoids and carotenoids [23], which may inhibit the oxidation of USFA. These substances may act as strong free radical scavengers: due to the presence of these plant-based active substances, the free radicals are converted into stable phenoxy radicals, thus terminating the free radical reaction chain [24, 25]. The peroxide value of FO was lower than SL in 0–8 days but higher than SL in 9–14 days (Figure 1). This may be because carotenoids and flavonoids have strong antioxidative activities and inhibit the oxidation of the oils.

### 3.4 Rheological properties

**Figure 2:**
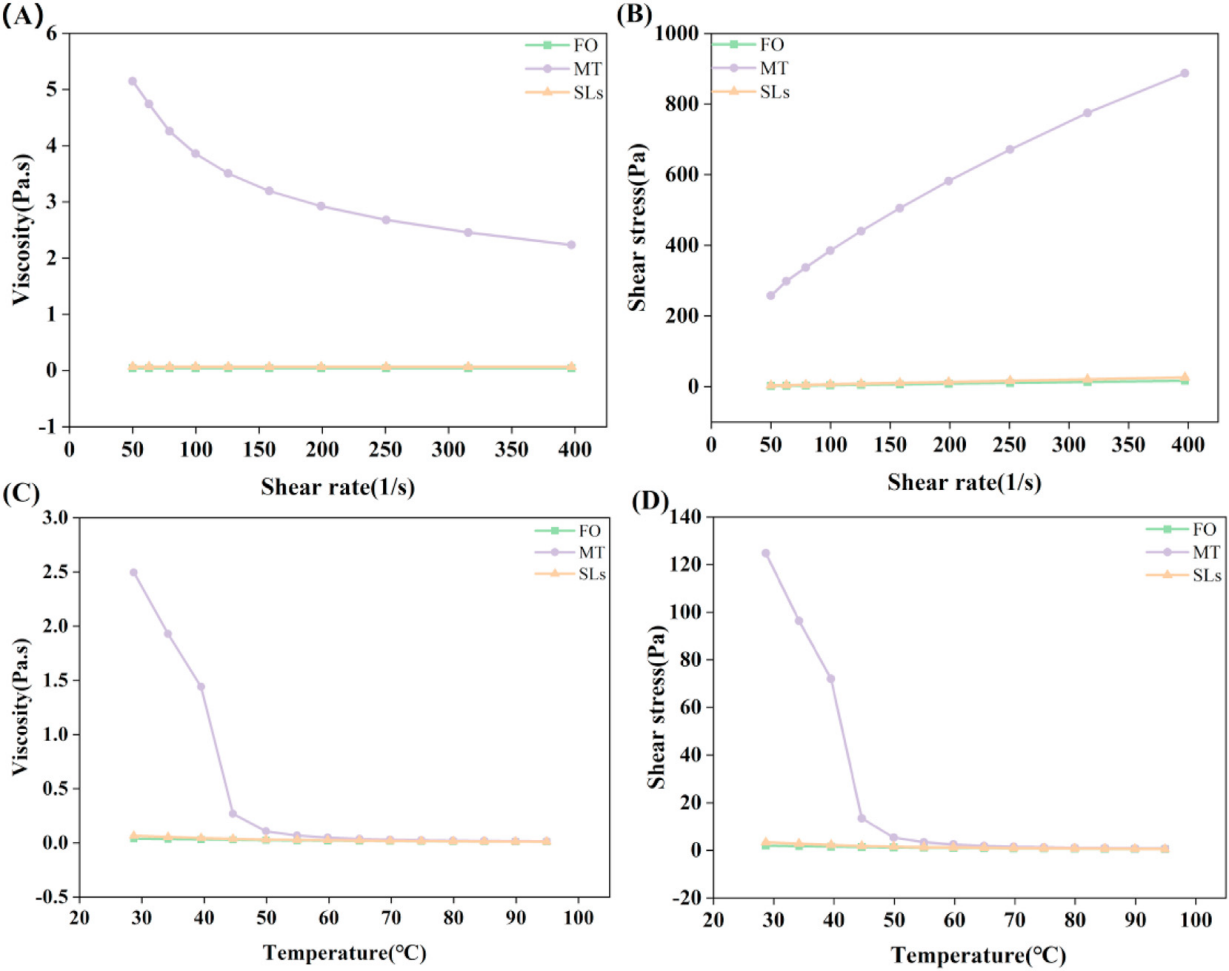
Effects of shear rate and temperature on apparent viscosity and shear stress of the three oils and fats. (A) the variation of viscosity at 55 °C and a shear rate range of 50s^−1^ to 400s^−1^; (B) the variation of shear force at 55 °C and a shear rate range of 50s^−1^ to 400s^−1^; (C) the variation of viscosity at a shear rate of 50s^−1^ and 55–95 °C, 5 °C/min heating; (D) the variation of shear force at a shear rate of 50s^−1^ and 55–95 °C, 5 °C/min heating.

As shown in Figure 2, FO and SL exhibited similar shear changes over the shear rate and temperature range tested and had significant differences with MT. The apparent viscosity of MT decreased with an increase of the shear rate, showing a non-Newtonian fluid property, and the viscosity of FO and SL did not change significantly (Figure 2a). The shears tress of FO, MT and SL increased with the increase of shear rate. It was particularly noteworthy that the shear stress of MT at 400 (1/s) was 52.7 and 33.1 times of FO and SL, respectively (Figure 2b). The apparent viscosity and shear stress of FO, MT and SL decreased with temperature increase, indicating shear dilution behavior (Figure 2c and d).

Indeed, the viscosity and shear force generally increased with the triacylglycerol FA chain length and declined with the degree of unsaturation. These results were consistent with previous reports [26, 27]. At a higher shear rate, the crimp of the molecular chain tends to be in the same direction because of the breakage of the molecular chain and the reduction of the side chain, thus reducing the viscosity. It was also reported that to disintegrate solids or increase deformation shear stress by increasing the shear rate, the oil and fat flow rate increased in a gradient [28]. As the shear rate raised, the shear stress of MT increased continuously, but the relationship was not linear, whereas the relationship between shear rate and shear stress of FO and transesterified SL was nearly linear. Based on this analysis, MT was a typical pseudoplastic non-newtonian fluid with a wide range of shear rates, while FO and SL were Newtonian fluids. The temperature was found to influence the rheological properties of MT significantly. Some of the primary FAs in oils and fats (C18:1 and C18:2) mainly affected their crystallization temperatures. A lower crystallization temperature was associated with a higher proportion of C18:2 and a lower percentage of C18:1.

Similarly, melting point was found to be highly corrected with viscosity, C18:1 and C18:2 content, and the ratio of SFA to USFA and MUFA to PUFA of three different oils and fats [30]. FAs with more double bonds do not have a rigid and fixed structure, with fluid-like behaviors. The apparent viscosity and shear force of MT decreased sharply near the melting point (46.2 ± 1.5 °C), which is because MT exhibits a solid structure at room temperature, and the stress required to destroy the molecular chain structure of fat solid is high. With the increase of temperature, the fat gradually transforms from solid-state to liquid state, and the crosslinking between long-chain FA is weakened, and the intermolecular interaction is reduced. Therefore, when the temperature of MT reaches the melting point, its apparent viscosity and shear stress tend to be stable. The more saturated the lipids, the greater the change in apparent viscosity and shear stress with temperature [31]. The increase of temperature may lead to the increased thermal motion of the molecules inside the oil and fat and the decreased intermolecular interaction force, and the volume of the oil and fat expands with the temperature increasing, resulting in the decreased molecular number in each volume and the increased fluidity of the oil and fat. These results suggest that the primary components (SFA/MUFA/PUFA) may significantly contribute to the oil and fat flow behaviors [32].

### 3.5 Volatile compounds

**Figure 3:**
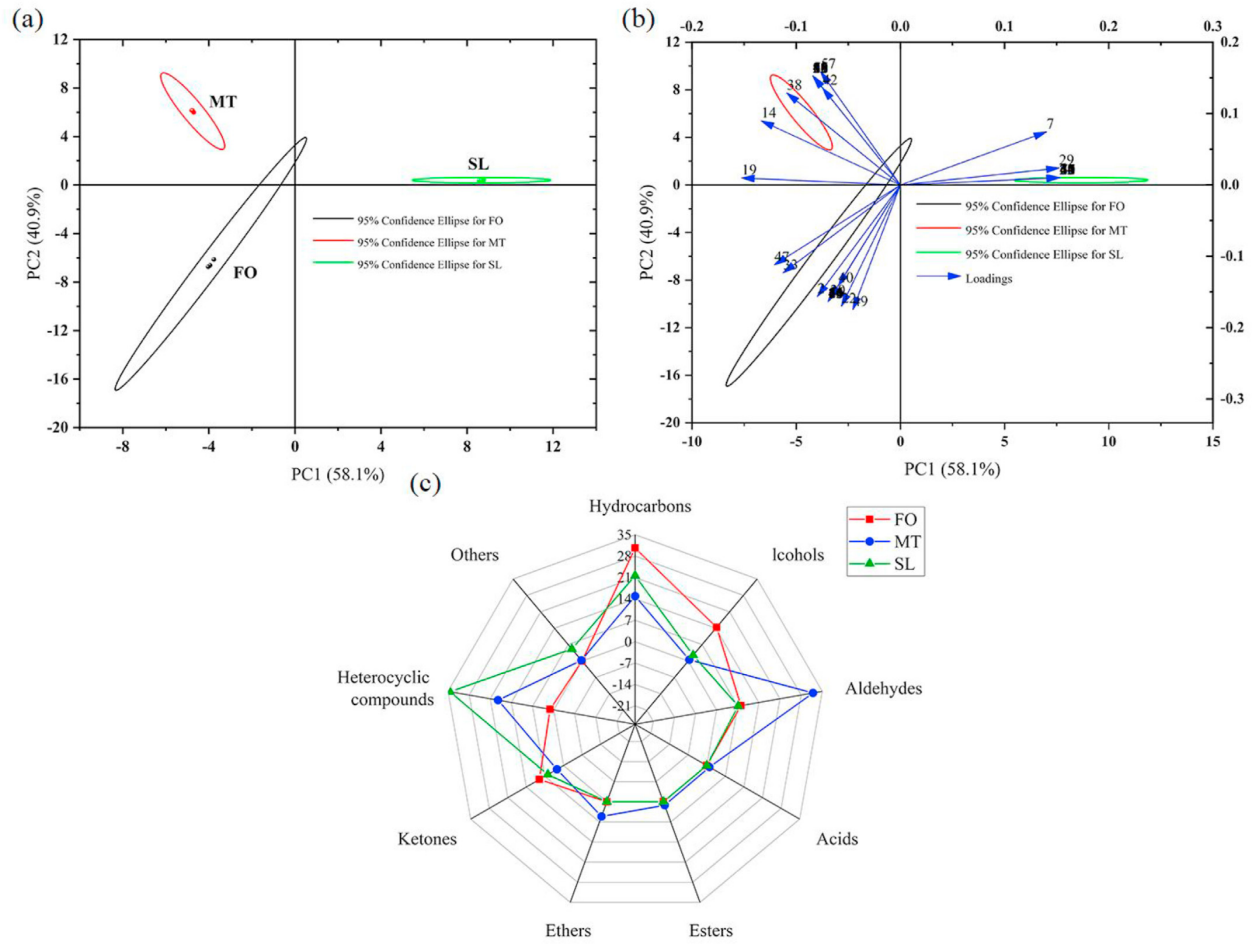
Principal components analysis (a), loading plots (b) and Mappa radar (c) of the three oils and fats by GC-MS analysis.

A total of 73 VCs were identified in the three samples (Table 3). A total of 24, 28 and 34 VCs were identified in FO, MT and SL, respectively. The VCs were classified into the following chemical groups: 20 hydrocarbons, 7 alcohols, 13 aldehydes, 13 ketones, 15 heterocyclic compounds, 1 acid (3-methylbutanoic acid), 1 ester (butyl acrylate) and 2 other compounds (Table 3). Five types of VCs were observed in FO, with high contents of hydrocarbons (31 ± 1%), alcohols (14.4 ± 0.1%) and ketones (9.2 ± 0.4%). All types of VCs were detected in MT, with high contents of aldehydes (32.0 ± 0.8%), heterocyclic compounds (18.5 ± 0.3%) and hydrocarbons (14.9 ± 0.2%). Five VCs were identified in SL, with high contents of heterocyclic compounds (34.4 ± 0.2%) and hydrocarbons (21.58 ± 0.08%). The total levels of VCs constituents in the three oils and fats varied greatly (Table 3, Figure 3c). Specifically, the most obvious difference was the increase of heterocyclic compounds in SL.

**Table 3:** Volatile compounds identified in the three oils using SPME-GC-MS.

Number	Compounds	FO			MT			SL		
		RT	RI	Similarity	RT	RI	Similarity	RT	RI	Similarity
**Hydrocarbons**										
1	2,3,3,4-Tetramethylpentane	ND	ND	ND	ND	ND	ND	3.900	3.89 ± 0.037	90
2	1,3-Cyclooctadiene	5.087	3.873 ± 0.061	85	6.492	0.333 ± 0.012	85	ND	ND	ND
3	1,3,5,7-Cyclooctatetraene	ND	ND	ND	5.683	3.177 ± 0.058	91	ND	ND	ND
4	5,10-Dioxabicyclodecane	5.650	6.083 ± 0.052	89	ND	ND	ND	ND	ND	ND
5	3-Oxatricyclo [3.2.1.02,4] octane,(1R,2S,4R,5S)-rel-	6.405	0.53 ± 0.016	88	ND	ND	ND	ND	ND	ND
6	1,3,3-Trimethyltricyclo [2.2.1.02,6]Heptane	ND	ND	ND	6.597	0.867 ± 0.034	91	ND	ND	ND
7	2,2,4,6,6-Pentamethylheptane	ND	ND	ND	7.796	2.737 ± 0.056	85	7.812	7.597 ± 0.16	97
8	1-Ethylcyclohexene	7.826	18.603 ± 1.103	92	ND	ND	ND	ND	ND	ND
9	Undecane	ND	ND	ND	7.986	1.243 ± 0.039	93	ND	ND	ND
10	(+)-Limonene	8.556	0.593 ± 0.025	90	ND	ND	ND	ND	ND	ND
11	2,2,4,4,6,8,8-Heptamethylnonane	ND	ND	ND	ND	ND	ND	8.620	0.687 ± 0.021	88
12	Limonene	ND	ND	ND	8.677	3.897 ± 0.012	91	ND	ND	ND
13	m-Cymene	ND	ND	ND	ND	ND	ND	9.903	4.587 ± 0.14	95
14	Dodecane	12.463	0.71 ± 0.024	93	10.152	1.547 ± 0.045	91	ND	ND	ND
15	2-Methyl-1-phenylpropene	ND	ND	ND	ND	ND	ND	10.596	4.51 ± 0.033	87
16	2-Decyloxirane	ND	ND	ND	12.292	0.497 ± 0.021	96	ND	ND	ND
17	2-Methyldecalin	ND	ND	ND	ND	ND	ND	12.992	0.313 ± 0.021	90
18	Tridecane	ND	ND	ND	14.973	0.34 ± 0.029	90	ND	ND	ND
19	Tetradecane	17.517	0.227 ± 0.017	95	17.522	0.257 ± 0.012	94	ND	ND	ND
20	2,6,11-Trimethyldodecane	29.358	0.027 ± 0.025	91	ND	ND	ND	ND	ND	ND
**Alcohols**										
21	1,7-Heptanediol	ND	ND	ND	ND	ND	ND	5.944	0.837 ± 0.029	87
22	1-Ethynyl-1-cyclohexanol	6.621	11.41 ± 0.136	87	ND	ND	ND	6.847	1.133 ± 0.029	93
23	4-Ethylcyclohexanol	7.657	2.387 ± 0.026	89	ND	ND	ND	ND	ND	ND
24	Trans-2-decen-1-ol	ND	ND	ND	8.459	0.537 ± 0.037	87	ND	ND	ND
25	(+)-Isomenthol	ND	ND	ND	ND	ND	ND	10.083	0.53 ± 0.051	96
26	2-Cyclohexylethanol	12.292	0.583 ± 0.026	91	ND	ND	ND	ND	ND	ND
27	4-tert-Butylbenzyl alcohol	ND	ND	ND	ND	ND	ND	13.166	0.583 ± 0.033	89
**Aldehydes**										
28	(2E)-2-Nonenal	ND	ND	ND	3.300	18.143 ± 0.805	94	ND	ND	ND
29	Hexanal	ND	ND	ND	11.634	0.317 ± 0.025	86	3.373	3.833 ± 0.061	92
30	Heptanal	ND	ND	ND	5.925	3.547 ± 0.092	95	ND	ND	ND
31	Heptenal	6.947	4.5 ± 0.054	87	ND	ND	ND	ND	ND	ND
32	3-Thiophenecarboxaldehyde	ND	ND	ND	ND	ND	ND	6.650	1.143 ± 0.053	86
33	(2E)-2-Octenal	9.204	2.143 ± 0.117	93	9.305	1.077 ± 0.045	88	7.152	0.483 ± 0.025	85
34	Benzaldehyde	ND	ND	ND	ND	ND	ND	7.302	0.483 ± 0.034	87
35	(E,E)-2,4-Heptadienal	ND	ND	ND	ND	ND	ND	7.986	0.813 ± 0.021	89
36	Octanal	ND	ND	ND	8.094	2.193 ± 0.119	94	ND	ND	ND
37	1-Propanol-2,2-dimethyl-benzoate	ND	ND	ND	ND	ND	ND	10.158	0.42 ± 0.014	91
38	Nonanal	10.239	1.387 ± 0.042	95	10.299	6.337 ± 0.09	96	ND	ND	ND
39	Decanal	ND	ND	ND	12.699	0.347 ± 0.025	92	ND	ND	ND
40	Pentadecanal	27.625	0.067 ± 0.034	87	ND	ND	ND	ND	ND	ND
**Acids**										
41	3-Methylbutanoic acid	ND	ND	ND	4.692	1.013 ± 0.029	92	ND	ND	ND
**Esters**										
42	Butyl acrylate	ND	ND	ND	5.833	1.183 ± 0.497	89	ND	ND	ND
**Ethers**										
43	Ether	ND	ND	ND	4.934	5.09 ± 0.067	93	ND	ND	ND
**Ketones**										
44	2-Heptanone	5.339	0.777 ± 0.045	87	ND	ND	ND	ND	ND	ND
45	4-Methylcyclohexanone	ND	ND	ND	ND	ND	ND	11.332	1.473 ± 0.046	86
46	2-Methyloctan-4-one	ND	ND	ND	ND	ND	ND	7.392	0.337 ± 0.025	92
47	6-Methylhept-5-en-2-one	7.533	2.137 ± 0.129	85	7.700	0.92 ± 0.045	94	ND	ND	ND
48	1,5-Cyclooctadien-4-one	9.698	0.833 ± 0.025	81	ND	ND	ND	ND	ND	ND
49	3,5-Octadiene-2-one	9.452	3.44 ± 0.184	90	ND	ND	ND	9.538	0.607 ± 0.012	89
50	(2E)3,5-octadiene-2-one	10.015	1.773 ± 0.034	92	ND	ND	ND	ND	ND	ND
51	2,4,6-Trimethoxyacetophenone	ND	ND	ND	ND	ND	ND	12.167	3.163 ± 0.053	91
52	Thujone	ND	ND	ND	ND	ND	ND	12.860	0.32 ± 0.008	88
53	Cycloheptadecanone	ND	ND	ND	17.575	0.873 ± 0.012	90	ND	ND	ND
54	2-Tridecanone	ND	ND	ND	19.971	0.417 ± 0.009	87	ND	ND	ND
55	Heptadecan-2-one	ND	ND	ND	24.891	0.267 ± 0.017	87	ND	ND	ND
56	Furaltadone	29.100	0.143 ± 0.021	89	ND	ND	ND	ND	ND	ND
**Heterocyclic compounds**										
57	p-Xylene	ND	ND	ND	5.170	17.847 ± 0.257	97	5.207	1.157 ± 0.012	87
58	Cumene	ND	ND	ND	ND	ND	ND	6.424	0.617 ± 0.012	93
59	Benethamine	ND	ND	ND	ND	ND	ND	7.050	0.393 ± 0.021	90
60	2-Propylfuran	ND	ND	ND	ND	ND	ND	8.314	0.987 ± 0.025	88
61	3-Aminopyrazole	8.717	0.84 ± 0.022	87	ND	ND	ND	ND	ND	ND
62	1,4-Diethylbenzene	ND	ND	ND	ND	ND	ND	9.274	16.127 ± 0.278	98
63	Dicyclopropyl methyl amine	9.792	0.343 ± 0.012	86	ND	ND	ND	ND	ND	ND
64	1-Ethyl-4-(2-methylpropyl)benzene	ND	ND	ND	ND	ND	ND	10.255	0.48 ± 0.033	91
65	2-Methyl-2,3-dihydro-1H-indene	ND	ND	ND	ND	ND	ND	10.841	0.693 ± 0.021	88
66	tert-Pentylbenzene	ND	ND	ND	ND	ND	ND	11.050	6.443 ± 0.077	87
67	1-Methylindan	ND	ND	ND	ND	ND	ND	11.208	0.667 ± 0.017	88
68	Methylindene	ND	ND	ND	ND	ND	ND	11.462	1.933 ± 0.045	89
69	(±)-Camphor	ND	ND	ND	11.463	0.607 ± 0.039	91	ND	ND	ND
70	1,4-Dihydronaphthalene	ND	ND	ND	ND	ND	ND	11.884	0.76 ± 0.029	95
71	Naphthalene	ND	ND	ND	ND	ND	ND	12.413	4.127 ± 0.021	97
**Others**										
72	[(E)-tetradec-9-enyl] acetate	ND	ND	ND	ND	ND	ND	12.012	5.12 ± 0.079	92
73	2-Chloro-8-(methylamino)	ND	ND	ND	20.042	0.287 ± 0.017	89	ND	ND	ND

RI = Retention indices, RT = Retention time. ND: Not detected.

Volatile compounds were identified by the NIST 17 library and were only reported when the similarity was greater than 85.

Most of the heterocyclic compounds are produced by the oxidative decomposition of lipids. Heterocyclic compounds as a portion of fat and fresh meat flavor have an important role in the sensory system, and the effect of transesterification on flavor compounds is beneficial [33, 34]. More volatile compounds, aldehydes were identified in MT than in FO and SL. Highly volatile compounds in FO, MT and SL identified were (2E)-2-nonenal, hexanal, heptanal, heptenal, 3-thiophenecarboxaldehyde, (2E)-2-octenal, benzaldehyde, (E, E)-2,4-heptadienal, octanal, nonanal, decanal and pentadecanal (Table 3). The autoxidation of aldehydes, mainly unsaturated fatty acids, is accompanied by the production of small molecule aldehydes via the Strecker degradation pathway. The essence of Strecker degradation is the oxidative decarboxylation of lipid-derived reactive carbonyl groups [35]. It has been reported that fats provided a flavor precursor, free FA, which was further degraded to produce aldehydes and Tan sheep's unique flavor [36]. MT contained 34.05% unsaturated FA (Table 2), but its peroxide value was higher than FO and MT (Table 1, Figure 1), indicating that its oxidation degree is higher, with higher contents of volatile aldehydes. The hydrocarbons identified included straight-chain alkanes, side-chain alkanes, cycloalkanes, olefins, alkynes and their configurational isomers. The appearance of hydrocarbons in the three oils is attributed to the free radical reaction of unsaturated and saturated fatty acyl chains. Although the contribution of hydrocarbons to flavor is small, the formation of hydrocarbons indirectly reflects the saturation of oil, and unsaturated FA is beneficial to the formation of hydrocarbons [37]. Alcohols are produced by the oxidative decomposition of FA and the degradation of linolenic acids. For example, 1,7-heptanediol might be the decomposition product of oleic acid. Volatile ketones were considered to be related to the flavor of the oil, and fats were common constituents of most of the oils and fat products [15]. Due to their typical odors and low perception thresholds in volatile compounds, ketones retained an unique mutton flavor. Only a small number of acids, esters, ethers and furan were detected, which may be related to the oil and fat composition. The Strecker degradation of the lipid-derived reactive carbonyls in oils is an oxidative decarboxylation reaction by which these compounds are transformed into decarboxylated deaminated carbonyl compounds in the presence of a variety of reagents under different reaction conditions [35]. To gain a comprehensive representation of the primary VCs that differentiated the three samples, a principal component analysis (PCA) was performed on the entire dataset. As shown in Figure 3a, PCA was conducted to determine the relevance among the distribution of VCs and different oils and fats. PC-1 and PC-2 expressed 58.1% and 40.9% of the variability of VCs, respectively. The score plot of the first two PCs showed a satisfactory transesterification degree of the FO, MT and SL in terms of VCs. As shown in Figure 3b, the variables influencing the identification of the three samples in terms of transesterification on the first two PCs were identified in the loading diagram. The primary VCs that were positively correlated with PC-1 were 2,2,4,6,6-pentamethylheptane (7), (+)-isomenthol (25), hexanal (29), benzaldehyde (34), (E,E)-2,4-heptadienal (35), 2-methyloctan-4-one (45), benethamine (58) and dicyclopropyl methylamine (62), and those that were positively correlated with PC-2 were 1,3,3-trimethyltricyclo[2.2.1.02,6] heptane (6), limonene (12), dodecane (14), tetradecane (19) and nonanal (38).

## 4 Conclusions

This study was undertaken to provide the lipid industry with information on the physicochemical properties of SL to improve its application as edible oil and fat and value-added food ingredient. The results showed that SL had the color and apparent viscosity similar to vegetable oils, low melting point and reasonable fatty acid ratio. The physicochemical indexes (acid value, peroxide value and saponification value) of SL were lower than the maximum values provided by Codex Alimentarius (2005), which met the safety standards of edible oils and fats. Future research should further explore the combination of various vegetable oils and animal fats and the selection of materials for other food ingredients to improve the safety of SL in the food industry. As the enzymatic transesterification formulation can be easily translated to large-scale production, this modification and reuse of animal fat may have great application potential in the food industry.

## Declarations

### Author contribution statement

Jun Liu: Conceived and designed the experiments; Performed the experiments; Analyzed and interpreted the data; Wrote the paper.

Weiyi Zhang: Conceived and designed the experiments; Performed the experiments.

Dunhua Liu: Conceived and designed the experiments; Performed the experiments; Contributed reagents, materials, analysis tools or data.

Wei Zhang: Conceived and designed the experiments; Contributed reagents, materials, analysis tools or data.

Lu Ma: Contributed reagents, materials, analysis tools or data.

Shuzhe Wang: Conceived and designed the experiments.

### Funding statement

This work was supported by Funds for Independent Innovation in Agricultural Science and Technology in Ningxia Hui Autonomous Region (Demonstration of Science and Technology Innovation for High-Quality Agricultural Development and Ecological Protection, NGSB-2021-6-05) and The national Spark Program of China (2015GA880005).

### Data availability statement

The data that has been used is confidential.

### Declaration of interests statement

The authors declare no conflict of interest.

### Additional information

No additional information is available for this paper.
